# Metagenome-assembled genomes from irradiated human dental plaque

**DOI:** 10.1128/mra.00917-24

**Published:** 2024-12-31

**Authors:** Romualdo M. Filho, Claudia Mengoni, Julia S. Bruno, Eduardo R. Fregnani, Nicola Segata, Anamaria A. Camargo, Vitor Heidrich

**Affiliations:** 1 Molecular Oncology Center, Hospital Sírio-Libanês 42522 , São Paulo, Brazil; 2 CIBIO, University of Trento 19034 , Trento, Italy; 3 Department of Experimental Oncology, IEO European Institute of Oncology IRCCS, Milan, Italy; 4 Department of Twins Research and Genetic Epidemiology, King’s College London, London, United Kingdom; Montana State University, Bozeman, Montana, USA

**Keywords:** oral microbiome, head and neck cancer, radiotherapy, metagenomics

## Abstract

We provide 309 quality-controlled bacterial metagenome-assembled genomes recovered from supragingival plaque metagenomes. Samples were collected from head and neck cancer patients following radiotherapy, so the recovered genomes can be useful to investigate the effects of oral cavity irradiation on oral microbiome members.

## ANNOUNCEMENT

Radiotherapy is a standard therapeutic approach for head and neck cancer (1), but this comes at the cost of treatment-related oral toxicities (2). Radiation-related caries is an aggressive painless form of dental caries that affects almost a third of irradiated head and neck cancer patients (3). Although its pathophysiology remains elusive, paralleling conventional caries (4), microbial participation in its onset and progression is likely. In a previous study (5), we collected and metagenomically sequenced supragingival plaque samples (*n* = 24) from head and neck cancer patients treated with radiotherapy (some of whom developed radiation-related caries). Following DNA extraction of plaque samples as previously described (6), metagenomic library preparation was performed with the Nextera-XT DNA Library Prep Kit (Illumina) and sequenced on the Illumina NovaSeq 6000 (2 × 150 bp), generating 734 M paired-end metagenomic reads. Following preprocessing with kneaddata version 0.7.7-alpha (https://github.com/biobakery/kneaddata) for the removal of low-quality and contaminating (human or phiX174) reads, resulting metagenomes had an average depth of 2.5 Gbp (range: 0.2–12.6 Gbp).

Considering that little is known about the potential genetic alterations induced and selected by radiotherapy on human microbiome members, we reconstructed microbial genomes from irradiated plaque metagenomes. Metagenomic assembly was performed with MEGAHIT version 1.1.1 (7), and after filtering of contigs shorter than 1,000 bp, binning was performed with MetaBAT2 version 2.12.1 (8) using the coverage information computed by mapping reads against contigs with BowTie2 version 2.2.9 (9). The resulting 659 metagenome-assembled genomes (MAGs) were quality checked with CheckM version 1.1.3 (using the *lineage_wf* option) (10), and MAGs with either completeness ≤50% or contamination >5% were tagged as low quality (*n* = 350). Considering the ubiquity of fungi such as *Candida albicans* in the human oral cavity (11), we evaluated the possibility that eukaryotic MAGs were among the low-quality set by running BUSCO version 5.6.1 (12). Since no eukaryotic MAGs were retrieved, all low-quality MAGs were discarded. The remaining 309 at least medium-quality prokaryotic MAGs are available through this Announcement.

Taxonomic classification of these MAGs according to GTDB-Tk version 2.3.2 (r207 database) (13) and PhyloPhlAn version 3.1 (vJun23 database) (14) is provided in Table 1, as well as genome characteristics (size and GC content), assembly metrics (number of contigs, *N*_50_, coverage, and CheckM completeness and contamination), and genomic features (number of coding sequences [CDSs], tRNAs, and rRNAs) as annotated using Prokka version 1.14.5 (15). All aforementioned tools were applied with default parameters. We also indicate in Table 1 whether the MAG was recovered from a metagenome from an individual who developed radiation-related caries. According to the widely used criteria (16), the resource spans 122 high-quality and 187 medium-quality MAGs, all of which were assigned to the domain Bacteria, with 9 bacterial phyla represented. Overall, genome size ranged between 376 and 3,422 kbp, and GC content ranged between 27.4% and 72.4%, with rRNAs retrieved for 57.3% of the MAGs. This resource will enable insights into the genomic characteristics of plaque microbes in the post-radiation oral environment and their potential role in biofilm-dependent diseases.

**TABLE 1:** Metagenome-assembled genomes characteristics^*a*^

MAG id	GTDB assignment (r207)	PhyloPhlAn assignment (vJun23)	Genome size (bp)	GC content (%)	No. of contigs	N50 contigs (bp)	Coverage (x)	CheckM completeness (%)	CheckM contamination (%)	No. of CDS	No. of tRNAs	No. of rRNAs	GenBank accession no. (assembly)	SRA accession no. (raw reads)	Radiation-related caries
450__bin.1	s__Limosilactobacillus fermentum	NA	587377	53.91	22	37645	28.5	50.00	0.00	602	4	0	JBHRRP000000000	SRR28645827	Yes
450__bin.4	s__Streptococcus halitosis	NA	1808858	41.44	50	48103	47.2	99.42	0.68	1757	31	0	JBHRRO000000000	SRR28645827	Yes
450__bin.7	s__Lacticaseibacillus rhamnosus	s__Lacticaseibacillus rhamnosus (SGB7144)	2865742	46.70	331	12654	14.9	98.46	2.21	2627	42	2	JBHRRN000000000	SRR28645827	Yes
456__bin.2	s__Streptococcus sp900766505	NA	1349987	42.62	487	2874	10.7	68.59	2.99	1266	13	0	JBHRRM000000000	SRR28645826	Yes
460__bin.2	s__Rothia dentocariosa	s__Rothia SGB49305 (SGB49305_group)	2135504	54.12	349	7585	9.9	83.53	0.00	1920	45	2	JBHRRL000000000	SRR28645815	Yes
460__bin.6	s__Actinomyces oris_C	s__Actinomyces oris (SGB15881)	3030277	68.50	284	17610	43.4	97.30	0.91	2514	48	3	JBHRRK000000000	SRR28645815	Yes
460__bin.8	s__Peptidiphaga sp000466165	s__Actinobaculum sp oral taxon 183 (SGB15892)	2240854	68.18	111	30916	48.9	98.33	1.81	1876	45	1	JBHRRJ000000000	SRR28645815	Yes
473__bin.12	g__UBA9732	s__GGB96320 SGB123478 (SGB123478)	1350750	46.08	298	5358	9.9	73.30	0.00	1226	32	1	JBHRRI000000000	SRR28645810	Yes
473__bin.14	s__Prevotella denticola	s__Prevotella denticola (SGB1540)	2342664	51.68	279	11021	50.0	87.79	3.86	1829	33	0	JBHRRH000000000	SRR28645810	Yes
473__bin.15	s__Anaeroglobus micronuciformis	s__Megasphaera micronuciformis (SGB5868)	1642084	45.62	233	9037	31.0	94.78	3.71	1557	37	1	JBHRRG000000000	SRR28645810	Yes
473__bin.16	s__Veillonella sp905214495	NA	1324908	39.33	121	16324	172.2	57.40	0.86	1219	22	0	JBHRRF000000000	SRR28645810	Yes
473__bin.17	s__Pauljensenia sp000185285	s__Schaalia meyeri (SGB17151)	2262155	66.53	183	16825	50.3	93.68	2.52	2063	47	1	JBHRRE000000000	SRR28645810	Yes
473__bin.19	s__Streptococcus gordonii	NA	1569046	40.63	129	20137	44.7	56.83	0.00	1478	7	0	JBHRRD000000000	SRR28645810	Yes
473__bin.2	s__Lancefieldella parvula	s__Lancefieldella parvula (SGB964)	1061864	45.43	220	5404	10.8	70.69	1.72	940	24	1	JBHRRC000000000	SRR28645810	Yes
473__bin.20	s__Mitsuokella sp000469545	s__Mitsuokella sp oral taxon 131 (SGB5901)	1820261	58.10	78	38142	27.5	70.33	0.55	1690	34	1	JBHRRB000000000	SRR28645810	Yes
473__bin.25	s__Prevotella oulorum	s__Prevotella oulorum (SGB1520)	2140285	47.62	150	18810	23.5	91.84	0.51	1771	43	1	JBHRRA000000000	SRR28645810	Yes
473__bin.26	s__Porphyromonas endodontalis	s__Porphyromonas endodontalis (SGB2016)	1855064	47.74	61	47572	18.6	97.50	0.47	1521	39	1	JBHRQZ000000000	SRR28645810	Yes
473__bin.27	s__Prevotella multisaccharivorax	s__Prevotella multisaccharivorax (SGB1702)	2903091	48.87	282	14276	20.1	96.59	3.05	2463	34	3	JBHRQY000000000	SRR28645810	Yes
473__bin.28	s__Propionibacterium acidifaciens	s__Propionibacterium acidifaciens (SGB15903)	2539063	71.83	270	13940	30.2	93.97	1.75	2213	40	0	JBHRQX000000000	SRR28645810	Yes
473__bin.3	s__Parascardovia denticolens	s__Parascardovia denticolens (SGB17291)	1590106	59.02	69	40065	36.8	92.34	0.23	1265	38	0	JBHRQW000000000	SRR28645810	Yes
473__bin.30	s__Metamycoplasma salivarium	s__Metamycoplasma salivarium (SGB5934)	376272	27.71	125	3137	10.3	56.50	0.85	659	13	0	JBHRQV000000000	SRR28645810	Yes
473__bin.33	s__Prevotella oris	s__Prevotella oris (SGB1525)	2588732	44.03	303	12098	33.7	94.39	4.18	2136	50	3	JBHRQU000000000	SRR28645810	Yes
473__bin.34	s__Campylobacter_A concisus_B	NA	1425529	38.23	389	4125	8.9	82.00	1.05	1345	13	0	JBHRQT000000000	SRR28645810	Yes
473__bin.36	g__Prevotella	s__GGB74353 SGB98242 (SGB98242)	1715255	49.05	401	4887	10.1	50.31	0.00	1464	17	0	JBHRQS000000000	SRR28645810	Yes
473__bin.37	s__Prevotella conceptionensis	NA	3247241	47.90	398	10825	25.0	93.18	0.65	2561	38	1	JBHRQR000000000	SRR28645810	Yes
473__bin.38	s__Selenomonas sp905373085	s__Selenomonas SGB5896 (SGB5896)	1586218	58.78	442	3962	9.9	69.03	1.37	1531	13	0	JBHRQQ000000000	SRR28645810	Yes
473__bin.39	s__Granulicatella adiacens	s__Granulicatella adiacens (SGB8249)	1602462	38.14	123	18506	20.4	85.69	0.94	1532	18	1	JBHRQP000000000	SRR28645810	Yes
473__bin.4	s__Streptococcus mutans	s__Streptococcus mutans (SGB8000)	2188099	36.68	150	29164	39.9	96.59	4.59	2092	33	1	JBHRQO000000000	SRR28645810	Yes
473__bin.40	s__Pauljensenia sp000308055	s__Actinomyces sp ph3 (SGB17137_group)	1831759	55.98	96	26905	19.8	97.65	1.54	1608	41	0	JBHRQN000000000	SRR28645810	Yes
473__bin.44	s__Rothia dentocariosa	s__Rothia SGB49305 (SGB49305_group)	1567211	55.08	351	5224	16.4	55.29	1.82	1413	29	1	JBHRQM000000000	SRR28645810	Yes
473__bin.49	s__Limosilactobacillus fermentum	s__Limosilactobacillus fermentum (SGB7106)	1250885	53.52	314	4822	9.4	82.49	2.46	1192	19	1	JBHRQL000000000	SRR28645810	Yes
473__bin.50	s__Leptotrichia wadei	s__Leptotrichia wadei (SGB6054)	2013045	29.96	436	5918	25.9	94.32	4.92	1849	25	0	JBHRQK000000000	SRR28645810	Yes
473__bin.51	s__Prevotella sp013333935	s__GGB1202 SGB1566 (SGB1566)	1877843	48.99	453	4794	10.8	72.65	3.56	1589	18	0	JBHRQJ000000000	SRR28645810	Yes
473__bin.54	s__Bifidobacterium dentium	s__Bifidobacterium dentium (SGB17234)	2113736	58.94	204	15079	13.1	92.69	1.44	1745	53	1	JBHRQI000000000	SRR28645810	Yes
473__bin.7	s__Stomatobaculum longum	s__GGB3883 SGB5265 (SGB5265)	1515265	56.53	363	5030	8.2	84.87	0.95	1398	24	0	JBHRQH000000000	SRR28645810	Yes
473__bin.8	s__Oribacterium sp000160135	s__Oribacterium sp oral taxon 078 (SGB7282)	1273736	56.11	463	2907	6.8	56.77	0.00	1164	16	0	JBHRQG000000000	SRR28645810	Yes
711__bin.1	g__Campylobacter_A	NA	1762359	46.51	382	5745	12.7	83.98	2.54	1713	17	0	JBHRQF000000000	SRR28645809	No
711__bin.10	s__Prevotella salivae	s__Prevotella salivae (SGB1522)	2581805	41.66	539	5947	10.6	86.13	1.75	2019	37	0	JBHRQE000000000	SRR28645809	No
711__bin.13	s__Kingella_A denitrificans	s__Kingella denitrificans (SGB9437)	1716423	55.44	209	10828	33.2	85.10	2.17	1771	34	0	JBHRQD000000000	SRR28645809	No
711__bin.14	g__Nanogingivalis	s__GGB12785 SGB19827 (SGB19827_group)	584565	35.63	60	14838	25.8	62.03	0.14	597	30	2	JBHRQC000000000	SRR28645809	No
711__bin.15	g__Cardiobacterium	s__Cardiobacterium SGB9419 (SGB9419)	2606411	59.53	274	13936	41.2	92.79	1.47	2474	41	0	JBHRQB000000000	SRR28645809	No
711__bin.16	s__Veillonella parvula_A	s__Veillonella parvula (SGB6939)	1665443	39.32	351	5785	19.5	87.73	1.38	1452	21	1	JBHRQA000000000	SRR28645809	No
711__bin.17	s__Porphyromonas pasteri	s__Porphyromonas bobii (SGB2043)	1691558	56.42	125	19926	52.1	77.33	0.79	1307	37	2	JBHRPZ000000000	SRR28645809	No
711__bin.18	s__SDRW01 sp007845485	s__TM7 phylum sp oral taxon 348 (SGB19880)	820119	38.37	38	28262	93.1	63.12	0.46	798	40	1	JBHRPY000000000	SRR28645809	No
711__bin.20	s__Centipeda artemidis	s__Selenomonas artemidis (SGB5878)	2235493	57.04	210	16509	161.3	97.85	3.51	2106	50	4	JBHRPX000000000	SRR28645809	No
711__bin.21	s__Rothia mucilaginosa	NA	1466596	60.15	418	3834	11.3	63.83	2.40	1234	22	0	JBHRPW000000000	SRR28645809	No
711__bin.22	g__Nanosyncoccus	s__GGB12761 SGB19791 (SGB19791)	681316	38.40	39	34952	22.2	76.86	0.00	635	44	2	JBHRPV000000000	SRR28645809	No
711__bin.27	s__F0058 sp000163695	s__Bacteroidetes oral taxon 274 (SGB1341)	1905154	43.67	201	12848	19.8	94.35	1.63	1695	38	1	JBHRPU000000000	SRR28645809	No
711__bin.3	s__Corynebacterium matruchotii	s__Corynebacterium matruchotii (SGB17007)	2629682	57.40	245	15401	15.9	92.23	1.07	2499	42	3	JBHRPT000000000	SRR28645809	No
711__bin.31	s__Prevotella oralis	s__Prevotella oralis (SGB1528)	2424522	45.08	165	21223	18.2	91.83	0.99	1934	34	1	JBHRPS000000000	SRR28645809	No
711__bin.33	s__Capnocytophaga gingivalis	s__Capnocytophaga gingivalis (SGB2479)	1932709	41.00	358	6746	41.6	83.41	0.48	1717	30	0	JBHRPR000000000	SRR28645809	No
711__bin.37	s__HOT-345 sp003260355	s__SR1 bacterium human oral taxon HOT 345 (SGB6893)	1075117	38.06	69	21240	59.5	70.64	3.37	1983	39	2	JBHRPQ000000000	SRR28645809	No
711__bin.38	s__Centipeda sp001717585	NA	2010624	56.28	328	8335	36.7	88.63	1.11	1992	30	0	JBHRPP000000000	SRR28645809	No
711__bin.4	g__JABCPE02	NA	1749591	36.93	368	6188	22.7	92.78	2.69	1636	21	1	JBHRPO000000000	SRR28645809	No
711__bin.40	s__Arachnia propionica	s__Arachnia propionica (SGB15897)	3041050	66.44	504	8399	15.4	91.34	3.98	2862	50	1	JBHRPN000000000	SRR28645809	No
711__bin.44	s__Capnocytophaga leadbetteri	NA	1717079	40.81	162	15065	79.5	62.49	0.95	1499	25	0	JBHRPM000000000	SRR28645809	No
711__bin.5	g__Granulicatella	NA	1631586	34.31	449	4224	14.5	80.83	4.37	1433	11	1	JBHRPL000000000	SRR28645809	No
711__bin.7	s__Abiotrophia defectiva	NA	1422118	46.88	344	4687	15.1	75.41	3.54	1255	12	2	JBHRPK000000000	SRR28645809	No
714__bin.1	s__Lacticaseibacillus rhamnosus	s__Lacticaseibacillus rhamnosus (SGB7144)	2899823	46.75	425	9774	44.7	93.36	3.07	2649	37	2	JBHRPJ000000000	SRR28645808	Yes
714__bin.10	s__Propionibacterium freudenreichii	s__Propionibacterium freudenreichii (SGB15904)	2156152	67.38	522	4870	9.6	88.32	3.92	2059	41	0	JBHRPI000000000	SRR28645808	Yes
714__bin.11	s__Veillonella parvula_A	s__Veillonella parvula (SGB6939)	1168342	40.04	377	3143	8.0	62.24	1.05	995	6	0	JBHRPH000000000	SRR28645808	Yes
714__bin.17	s__Prevotella melaninogenica_B	NA	2535952	41.43	571	5278	11.1	84.78	2.54	1869	24	0	JBHRPG000000000	SRR28645808	Yes
714__bin.19	s__Limosilactobacillus oris	s__Limosilactobacillus oris (SGB7093)	1695252	50.93	177	13722	21.4	89.30	1.66	1555	31	0	JBHRPF000000000	SRR28645808	Yes
714__bin.2	s__Porphyromonas asaccharolytica	s__Porphyromonas asaccharolytica (SGB1981)	1009011	54.07	341	3002	6.8	62.85	1.72	801	15	0	JBHRPE000000000	SRR28645808	Yes
714__bin.20	g__Lactobacillus	NA	1045608	48.76	39	38535	18.4	89.17	0.92	1039	20	0	JBHRPD000000000	SRR28645808	Yes
714__bin.23	s__Lactobacillus crispatus	NA	896321	36.23	20	89675	197.8	53.45	0.00	862	7	0	JBHRPC000000000	SRR28645808	Yes
714__bin.24	s__Parascardovia denticolens	s__Parascardovia denticolens (SGB17291)	1222404	59.77	329	4066	8.7	73.71	1.75	1082	20	0	JBHRPB000000000	SRR28645808	Yes
714__bin.26	s__Olsenella_F profusa_A	s__Olsenella profusa (SGB14387)	2428193	64.12	193	19196	15.9	98.58	1.61	2253	49	1	JBHRPA000000000	SRR28645808	Yes
714__bin.4	s__Limosilactobacillus fermentum	s__Limosilactobacillus fermentum (SGB7106)	1588913	53.69	253	9058	15.6	94.91	2.68	1532	22	0	JBHROZ000000000	SRR28645808	Yes
714__bin.5	s__Limosilactobacillus vaginalis	s__Limosilactobacillus vaginalis (SGB7098)	1801075	40.79	121	28327	59.2	97.55	3.40	1792	28	2	JBHROY000000000	SRR28645808	Yes
714__bin.6	s__Scardovia wiggsiae	s__Scardovia wiggsiae (SGB15494)	1508835	52.88	41	52232	41.7	92.25	0.15	1236	45	0	JBHROX000000000	SRR28645808	Yes
714__bin.7	s__Bifidobacterium dentium	s__Bifidobacterium dentium (SGB17234)	2385426	58.88	201	15895	12.9	98.24	2.58	2024	48	2	JBHROW000000000	SRR28645808	Yes
714__bin.9	s__Prevotella oris	s__Prevotella oris (SGB1525)	2729643	43.97	236	16533	16.6	97.23	1.10	2246	41	2	JBHROV000000000	SRR28645808	Yes
722__bin.3	s__Neisseria subflava_C	s__Neisseria subflava (SGB9450_group)	1377536	50.19	452	3301	6.6	65.45	0.50	1460	23	1	JBHROU000000000	SRR28645807	Yes
722__bin.4	s__Pauljensenia sp000185285	s__Schaalia meyeri (SGB17151)	1401605	66.28	502	2858	6.6	57.84	1.15	1344	24	0	JBHROT000000000	SRR28645807	Yes
722__bin.5	s__Veillonella parvula_A	s__Veillonella parvula (SGB6939)	1672883	39.47	394	4974	14.5	90.42	1.49	1485	15	1	JBHROS000000000	SRR28645807	Yes
726__bin.10	s__Veillonella parvula_A	s__Veillonella parvula (SGB6939)	1789436	39.30	456	4340	16.5	87.11	4.76	1548	20	0	JBHROR000000000	SRR28645806	Yes
726__bin.12	s__Propionibacterium acidifaciens	s__Propionibacterium acidifaciens (SGB15903)	2375781	71.54	489	5877	12.6	86.34	1.29	2063	43	0	JBHROQ000000000	SRR28645806	Yes
726__bin.13	s__UBA7748 sp003862175	s__Atopobium sp oral taxon 416 (SGB94363)	2015734	55.38	206	14413	30.9	93.55	0.81	1903	41	1	JBHROP000000000	SRR28645806	Yes
726__bin.4	s__Prevotella oris	s__Prevotella oris (SGB1525)	2245065	44.36	727	3308	8.6	74.14	4.47	1950	28	0	JBHROO000000000	SRR28645806	Yes
726__bin.6	g__Eubacterium_T	NA	1597599	50.34	527	3163	6.3	68.49	0.71	1474	18	0	JBHRON000000000	SRR28645806	Yes
726__bin.8	s__Prevotella denticola	s__Prevotella denticola (SGB1540)	2418359	51.65	342	9485	10.9	92.85	1.26	1892	31	1	JBHROM000000000	SRR28645806	Yes
726__bin.9	s__Rothia dentocariosa	s__Rothia SGB49305 (SGB49305_group)	1842934	54.20	529	3762	8.8	78.85	2.74	1720	37	2	JBHROL000000000	SRR28645806	Yes
749__bin.11	s__Prevotella histicola	s__Prevotella histicola (SGB1543)	2548159	41.61	572	5401	9.3	89.81	3.34	1996	33	0	JBHROK000000000	SRR28645805	Yes
749__bin.12	s__Streptococcus mutans	s__Streptococcus mutans (SGB8000)	1918202	36.91	98	28492	22.7	97.66	0.95	1835	27	1	JBHROJ000000000	SRR28645805	Yes
749__bin.13	s__Scardovia wiggsiae	s__Scardovia wiggsiae (SGB15494)	1461217	53.34	53	43988	17.7	94.94	0.53	1181	41	0	JBHROI000000000	SRR28645805	Yes
749__bin.14	s__Parascardovia denticolens	s__Parascardovia denticolens (SGB17291)	1723266	58.94	52	48549	49.6	98.83	0.00	1382	44	1	JBHROH000000000	SRR28645805	Yes
749__bin.16	s__Limosilactobacillus vaginalis	s__Limosilactobacillus vaginalis (SGB7098)	1558444	40.92	159	17102	12.6	95.74	1.48	1467	24	0	JBHROG000000000	SRR28645805	Yes
749__bin.17	s__Bifidobacterium dentium	s__Bifidobacterium dentium (SGB17234)	2212894	58.89	320	9751	10.5	90.02	2.82	1928	47	1	JBHROF000000000	SRR28645805	Yes
749__bin.18	s__Actinomyces sp000220835	s__Actinomyces sp oral taxon 448 (SGB15872)	2300091	69.53	608	4379	11.7	81.49	2.68	2093	44	2	JBHROE000000000	SRR28645805	Yes
749__bin.2	s__Mitsuokella sp000469545	s__Mitsuokella sp oral taxon 131 (SGB5901)	1989129	57.65	165	17899	11.5	80.33	0.31	1849	34	1	JBHROD000000000	SRR28645805	Yes
749__bin.3	s__Propionibacterium acidifaciens	s__Propionibacterium acidifaciens (SGB15903)	2889322	71.24	194	21512	52.8	97.15	0.33	2501	48	3	JBHROC000000000	SRR28645805	Yes
749__bin.6	s__Veillonella parvula_A	s__Veillonella parvula (SGB6939)	1955708	38.94	189	15136	18.6	97.49	1.80	1794	35	1	JBHROB000000000	SRR28645805	Yes
749__bin.7	s__Limosilactobacillus fermentum	s__Limosilactobacillus fermentum (SGB7106)	865608	53.34	288	3148	6.7	63.80	0.82	800	20	0	JBHROA000000000	SRR28645805	Yes
749__bin.8	s__Lancefieldella parvula	s__Lancefieldella parvula (SGB964)	1413394	45.76	209	8402	11.0	95.30	1.55	1218	42	0	JBHRNZ000000000	SRR28645805	Yes
755__bin.1	s__Peptidiphaga gingivicola	s__Peptidiphaga gingivicola (SGB15894)	1587021	64.12	523	3223	7.8	62.47	2.08	1514	30	0	JBHRNY000000000	SRR28645804	No
755__bin.20	s__Rothia dentocariosa	s__Rothia SGB49305 (SGB49305_group)	1065657	51.81	30	83615	33.9	65.52	0.00	908	25	1	JBHRNX000000000	SRR28645804	No
755__bin.21	s__Corynebacterium matruchotii	s__Corynebacterium matruchotii (SGB17007)	2793494	57.26	113	37372	46.7	97.97	0.00	2526	47	4	JBHRNW000000000	SRR28645804	No
755__bin.22	s__Capnocytophaga granulosa	s__Capnocytophaga granulosa (SGB2483)	1346526	43.17	493	2792	8.9	51.27	2.17	1159	8	0	JBHRNV000000000	SRR28645804	No
755__bin.24	g__Selenomonas	s__Selenomonas SGB5897 (SGB5897_group)	1761046	58.08	410	5350	9.7	80.52	3.06	1654	30	1	JBHRNU000000000	SRR28645804	No
755__bin.27	s__Prevotella salivae	s__Prevotella salivae (SGB1522)	2808296	41.47	302	13870	21.5	92.44	1.93	2267	51	0	JBHRNT000000000	SRR28645804	No
755__bin.3	s__Lancefieldella rimae	NA	1015313	49.49	299	3822	7.0	57.11	0.07	940	21	1	JBHRNS000000000	SRR28645804	No
755__bin.6	s__Porphyromonas pasteri	s__Porphyromonas bobii (SGB2043)	1209789	56.67	408	3094	6.9	62.91	1.26	1042	28	2	JBHRNR000000000	SRR28645804	No
755__bin.7	s__Prevotella histicola	s__Prevotella histicola (SGB1543)	2666606	41.44	241	14894	64.3	93.92	2.08	2127	49	1	JBHRNQ000000000	SRR28645804	No
758__bin.17	s__Eikenella corrodens	s__Eikenella corrodens (SGB9434_group)	2043199	56.22	203	14079	29.2	90.89	3.14	1988	39	1	JBHRNP000000000	SRR28645825	No
758__bin.18	s__Corynebacterium matruchotii	s__Corynebacterium matruchotii (SGB17007)	2572822	57.27	343	10454	12.7	89.52	0.87	2488	34	1	JBHRNO000000000	SRR28645825	No
758__bin.19	g__Saccharimonas	NA	742697	48.31	28	40977	30.9	60.80	1.03	734	39	1	JBHRNN000000000	SRR28645825	No
758__bin.20	s__Alloprevotella tannerae	s__Alloprevotella SGB77721 (SGB77721_group)	2296317	46.48	234	13236	16.7	97.77	0.00	1875	41	2	JBHRNM000000000	SRR28645825	No
758__bin.25	s__Treponema sp010365865	s__Treponema vincentii (SGB3607)	2664273	44.82	422	8380	16.8	100.00	2.52	2375	48	2	JBHRNL000000000	SRR28645825	No
758__bin.26	s__Capnocytophaga granulosa	s__Capnocytophaga granulosa (SGB2483)	1895535	41.57	483	4306	77.0	58.15	2.59	1735	24	1	JBHRNK000000000	SRR28645825	No
758__bin.27	s__SDRW01 sp007845485	s__TM7 phylum sp oral taxon 348 (SGB19880)	750297	38.00	56	19036	20.5	63.73	0.00	720	42	0	JBHRNJ000000000	SRR28645825	No
758__bin.28	g__CAJPSE01	NA	1275742	62.38	484	2718	6.6	52.65	1.69	1264	15	1	JBHRNI000000000	SRR28645825	No
758__bin.3	s__Treponema_C lecithinolyticum	s__GGB2663 SGB3587 (SGB3587)	2215861	44.09	87	44334	17.4	98.60	0.02	1956	44	2	JBHRNH000000000	SRR28645825	No
758__bin.32	g__Treponema_D	s__GGB2666 SGB3592 (SGB3592)	1771976	42.00	578	3414	7.8	73.28	3.87	1518	30	1	JBHRNG000000000	SRR28645825	No
758__bin.34	s__Nanosynbacter sp013100825	NA	753678	50.94	26	39083	32.1	63.68	0.85	786	42	2	JBHRNF000000000	SRR28645825	No
758__bin.38	s__Porphyromonas sp000467855	s__Porphyromonas sp oral taxon 278 (SGB2041)	1129995	56.48	385	3035	6.8	60.80	1.62	924	12	0	JBHRNE000000000	SRR28645825	No
758__bin.40	s__F0058 sp905372605	s__Bacteroidota unclassified SGB1343 (SGB1343)	1877189	43.64	86	30699	22.7	97.31	1.11	1596	43	4	JBHRND000000000	SRR28645825	No
758__bin.44	s__Porphyromonas endodontalis	s__Porphyromonas endodontalis (SGB2016)	1912543	47.61	44	70672	24.9	99.61	0.00	1591	43	2	JBHRNC000000000	SRR28645825	No
758__bin.45	s__Capnocytophaga gingivalis	s__Capnocytophaga gingivalis (SGB2479)	1593136	41.45	373	4971	35.2	55.82	0.00	1372	23	1	JBHRNB000000000	SRR28645825	No
758__bin.46	s__Ottowia sp001262075	s__Ottowia sp Marseille P4747 (SGB12664)	2751721	63.99	182	22670	79.6	97.05	1.90	2434	43	0	JBHRNA000000000	SRR28645825	No
758__bin.5	s__Bacteroides_D sp013333835	s__GGB1025 SGB1319 (SGB1319)	2371847	55.48	167	20419	68.8	94.29	0.48	1768	38	0	JBHRMZ000000000	SRR28645825	No
758__bin.7	s__Campylobacter_A sp905371695	s__GGB12443 SGB19313 (SGB19313)	1552250	46.42	233	8809	22.2	86.68	3.72	1542	19	0	JBHRMY000000000	SRR28645825	No
758__bin.8	g__Nanoperiomorbus	NA	706532	51.13	108	7769	9.7	59.64	0.85	631	38	0	JBHRMX000000000	SRR28645825	No
761__bin.1	s__Corynebacterium durum	s__Corynebacterium durum (SGB17008)	2665812	57.27	366	9691	12.1	92.04	3.50	2629	47	0	JBHRMW000000000	SRR28645824	No
761__bin.10	s__F0058 sp000163695	s__Bacteroidetes oral taxon 274 (SGB1341)	1661529	44.05	416	4528	11.3	85.88	1.09	1459	31	3	JBHRMV000000000	SRR28645824	No
761__bin.12	s__Actinomyces massiliensis	s__Actinomyces massiliensis (SGB15873)	2256237	66.91	419	6931	31.5	65.79	3.66	2095	42	1	JBHRMU000000000	SRR28645824	No
761__bin.13	s__Rothia dentocariosa	s__Rothia SGB49305 (SGB49305_group)	1894778	54.12	490	4478	64.3	83.44	3.05	1769	43	0	JBHRMT000000000	SRR28645824	No
761__bin.3	g__Kingella	s__GGB6688 SGB9473 (SGB9473)	1128833	40.81	373	3204	7.6	69.62	1.22	1054	19	0	JBHRMS000000000	SRR28645824	No
761__bin.5	s__Veillonella parvula_A	s__Veillonella parvula (SGB6939)	1507804	39.68	426	3871	12.5	83.29	0.79	1300	9	2	JBHRMR000000000	SRR28645824	No
761__bin.7	s__Haemophilus_D parainfluenzae	s__Haemophilus parainfluenzae (SGB9712)	1738410	39.67	541	3461	12.7	82.89	4.89	1590	24	1	JBHRMQ000000000	SRR28645824	No
761__bin.8	s__Streptococcus sanguinis	s__Streptococcus sanguinis (SGB8050_group)	1970449	43.73	234	11906	18.1	72.57	2.07	1876	17	0	JBHRMP000000000	SRR28645824	No
761__bin.9	s__Corynebacterium matruchotii	s__Corynebacterium matruchotii (SGB17007)	1657050	57.75	502	3615	8.1	62.20	1.74	1719	24	1	JBHRMO000000000	SRR28645824	No
764__bin.3	s__Prevotella oris	s__Prevotella oris (SGB1525)	2457146	44.21	474	6885	9.4	91.29	0.40	2048	41	1	JBHRMN000000000	SRR28645823	No
764__bin.4	s__Actinomyces oris	s__Actinomyces oris (SGB15878)	1653366	68.64	683	2424	8.8	52.51	0.99	1508	30	0	JBHRMM000000000	SRR28645823	No
764__bin.5	s__Pauljensenia sp000185285	NA	1573146	66.16	517	3254	7.3	68.96	4.05	1566	23	1	JBHRML000000000	SRR28645823	No
764__bin.6	s__Corynebacterium matruchotii	s__Corynebacterium matruchotii (SGB17007)	1816200	57.62	546	3670	7.0	70.50	1.21	1897	33	1	JBHRMK000000000	SRR28645823	No
764__bin.7	s__Rothia dentocariosa	s__Rothia SGB49305 (SGB49305_group)	2243524	54.06	529	5144	19.2	87.97	4.75	2112	45	2	JBHRMJ000000000	SRR28645823	No
778__bin.10	s__Haemophilus seminalis	s__Haemophilus haemolyticus (SGB9649)	1805273	38.30	292	8270	53.1	66.14	0.00	1758	30	2	JBHRMI000000000	SRR28645822	No
778__bin.12	s__Actinomyces oris	s__Actinomyces oris (SGB15878)	2872127	68.83	354	11711	19.9	96.77	1.10	2405	49	2	JBHRMH000000000	SRR28645822	No
778__bin.15	s__Capnocytophaga ochracea	s__Capnocytophaga ochracea (SGB2497)	1549869	41.01	534	3020	8.2	58.80	0.19	1152	8	0	JBHRMG000000000	SRR28645822	No
778__bin.17	s__Neisseria elongata	s__Neisseria elongata (SGB9447)	1287401	55.38	403	3417	15.3	54.38	1.07	1352	18	0	JBHRMF000000000	SRR28645822	No
778__bin.19	g__Campylobacter_B	NA	1718526	48.06	431	4639	10.3	81.86	2.51	1554	21	0	JBHRME000000000	SRR28645822	No
778__bin.2	s__Prevotella denticola	s__Prevotella denticola (SGB1540)	2142570	51.67	382	7118	13.9	65.60	0.86	1704	18	0	JBHRMD000000000	SRR28645822	No
778__bin.20	s__Porphyromonas pasteri	s__Porphyromonas bobii (SGB2043)	2034677	55.96	207	12401	13.8	94.14	0.39	1582	43	1	JBHRMC000000000	SRR28645822	No
778__bin.21	s__Neisseria sicca_C	s__Neisseria sicca (SGB9465_group)	2369345	51.71	123	27631	72.5	96.53	2.92	2178	38	0	JBHRMB000000000	SRR28645822	No
778__bin.22	s__Veillonella parvula_A	s__Veillonella parvula (SGB6939)	2000126	38.86	134	24748	84.6	97.60	1.05	1814	39	3	JBHRMA000000000	SRR28645822	No
778__bin.3	s__Prevotella melaninogenica_B	NA	2927578	41.01	336	12218	39.9	93.44	1.07	2272	38	3	JBHRLZ000000000	SRR28645822	No
778__bin.4	s__Lautropia mirabilis	s__Lautropia mirabilis (SGB13165)	2500515	65.39	664	4350	8.1	81.47	1.97	2197	43	0	JBHRLY000000000	SRR28645822	No
778__bin.7	s__Centipeda noxia	s__Selenomonas noxia (SGB5884)	1892351	56.40	81	34771	20.0	84.69	0.96	1781	40	2	JBHRLX000000000	SRR28645822	No
778__bin.9	s__Prevotella oris	s__Prevotella oris (SGB1525)	2817936	43.84	188	23709	26.2	97.85	1.95	2331	46	1	JBHRLW000000000	SRR28645822	No
782__bin.1	g__Cardiobacterium	s__Cardiobacterium SGB9419 (SGB9419)	1941851	60.39	536	4156	10.7	79.35	1.32	1896	28	0	JBHRLV000000000	SRR28645821	Yes
782__bin.10	s__Leptotrichia hofstadii	s__Leptotrichia hofstadii (SGB6062)	1670366	32.34	545	3275	23.1	74.77	2.27	1458	26	0	JBHRLU000000000	SRR28645821	Yes
782__bin.12	s__Campylobacter_A sp905372745	s__Campylobacter SGB19316 (SGB19316_group)	2025526	46.16	282	9795	18.7	96.48	2.63	1880	31	0	JBHRLT000000000	SRR28645821	Yes
782__bin.13	s__Corynebacterium matruchotii	s__Corynebacterium matruchotii (SGB17007)	2776835	57.25	50	93969	244.0	96.05	0.06	2531	42	5	JBHRLS000000000	SRR28645821	Yes
782__bin.14	s__Lautropia mirabilis	s__Lautropia mirabilis (SGB13165)	2940210	65.67	314	13092	14.6	93.11	0.12	2462	38	0	JBHRLR000000000	SRR28645821	Yes
782__bin.15	s__Lachnoanaerobaculum saburreum	s__Lachnoanaerobaculum saburreum (SGB4481)	2433727	37.03	573	5159	14.7	93.99	2.76	2156	22	0	JBHRLQ000000000	SRR28645821	Yes
782__bin.16	s__Porphyromonas pasteri	s__Porphyromonas bobii (SGB2043)	2031303	55.94	267	9544	11.7	96.36	0.49	1583	48	1	JBHRLP000000000	SRR28645821	Yes
782__bin.17	s__Capnocytophaga ochracea	s__Capnocytophaga ochracea (SGB2497)	2337722	39.74	473	6093	34.5	90.87	3.93	1971	34	0	JBHRLO000000000	SRR28645821	Yes
782__bin.19	s__Haemophilus_D parainfluenzae	s__Haemophilus parainfluenzae (SGB9712)	1306714	39.70	444	3092	12.0	63.01	4.38	1200	10	0	JBHRLN000000000	SRR28645821	Yes
782__bin.20	s__Tannerella sp003033925	s__Tannerella serpentiformis (SGB2048)	1632240	58.50	408	4418	9.1	70.94	1.42	1335	22	0	JBHRLM000000000	SRR28645821	Yes
782__bin.25	g__Saccharimonas	s__Candidatus Nanosynsacchari sp TM7 ANC 38 39 G1 1 (SGB19882)	684207	48.48	70	12575	10.8	57.65	0.93	670	33	1	JBHRLL000000000	SRR28645821	Yes
782__bin.26	s__Veillonella parvula_A	s__Veillonella parvula (SGB6939)	1475028	38.83	382	4182	24.5	62.07	1.72	1342	24	0	JBHRLK000000000	SRR28645821	Yes
782__bin.27	s__Centipeda noxia	s__Selenomonas noxia (SGB5884)	1887537	56.62	102	29938	65.7	86.85	2.83	1818	47	3	JBHRLJ000000000	SRR28645821	Yes
782__bin.3	s__Aggregatibacter sp000466335	NA	1967970	43.14	207	14374	18.7	96.47	2.17	1879	45	4	JBHRLI000000000	SRR28645821	Yes
782__bin.30	s__Rodentibacter sp901420285	s__GGB6813 SGB9727 (SGB9727)	1377482	44.63	332	4719	10.9	78.63	2.02	1261	22	0	JBHRLH000000000	SRR28645821	Yes
782__bin.31	s__SDRW01 sp007845485	s__TM7 phylum sp oral taxon 348 (SGB19880)	618976	37.33	70	14439	19.6	54.63	0.93	595	28	1	JBHRLG000000000	SRR28645821	Yes
782__bin.32	g__Prevotella	NA	3168235	48.12	444	9113	14.1	92.24	0.68	2445	37	2	JBHRLF000000000	SRR28645821	Yes
782__bin.34	s__Pauljensenia odontolytica_A	s__Schaalia sp ORNL0103 (SGB17163_group)	2196495	65.33	337	9168	16.2	90.22	3.29	1988	40	1	JBHRLE000000000	SRR28645821	Yes
782__bin.6	s__Kingella_B oralis	s__Kingella bonacorsii (SGB9422)	1319294	54.88	469	2880	13.0	56.04	2.71	1333	27	0	JBHRLD000000000	SRR28645821	Yes
782__bin.7	s__Capnocytophaga granulosa	s__Capnocytophaga granulosa (SGB2483)	2486997	41.79	281	12121	70.6	94.76	1.72	2330	41	2	JBHRLC000000000	SRR28645821	Yes
785__bin.1	s__Neisseria sp000090875	s__Neisseria sp oral taxon 014 (SGB9463)	2298675	53.57	198	16809	13.8	96.41	1.35	2121	47	0	JBHRLB000000000	SRR28645820	No
785__bin.11	s__Lautropia mirabilis	NA	2985310	65.38	434	9519	10.7	93.60	0.16	2519	36	0	JBHRLA000000000	SRR28645820	No
785__bin.2	s__Campylobacter_A curvus	NA	1419113	45.56	420	3750	7.4	76.88	1.48	1386	11	0	JBHRKZ000000000	SRR28645820	No
785__bin.3	s__Rothia dentocariosa	s__Rothia SGB49305 (SGB49305_group)	2094949	53.93	547	4315	24.8	85.42	3.20	2003	38	1	JBHRKY000000000	SRR28645820	No
785__bin.4	s__Capnocytophaga endodontalis	s__Capnocytophaga genosp AHN8471 (SGB2498)	2449115	39.83	740	3544	11.0	75.72	4.90	2029	17	0	JBHRKX000000000	SRR28645820	No
785__bin.6	s__Capnocytophaga granulosa	s__Capnocytophaga granulosa (SGB2483)	1843574	43.36	510	4127	14.2	80.13	1.43	1529	23	2	JBHRKW000000000	SRR28645820	No
785__bin.8	s__Peptidiphaga sp000466165	s__Actinobaculum sp oral taxon 183 (SGB15892)	2245232	67.93	132	24640	30.9	99.17	1.33	1895	45	0	JBHRKV000000000	SRR28645820	No
785__bin.9	s__Veillonella parvula_A	s__Veillonella parvula (SGB6939)	1803271	39.45	393	5579	21.9	94.49	1.32	1613	24	0	JBHRKU000000000	SRR28645820	No
788__bin.12	g__Nanoperiomorbus	NA	764555	50.80	92	11071	11.8	59.54	2.56	672	39	1	JBHRKT000000000	SRR28645819	No
788__bin.14	s__Capnocytophaga ochracea	s__Capnocytophaga ochracea (SGB2497)	2361505	39.80	652	4042	26.3	84.90	3.40	1992	34	0	JBHRKS000000000	SRR28645819	No
788__bin.15	s__Scardovia wiggsiae	s__Scardovia wiggsiae (SGB15494)	1370491	53.43	208	8683	11.3	89.65	1.55	1143	42	0	JBHRKR000000000	SRR28645819	No
788__bin.2	g__Prevotella	NA	2321112	48.73	759	3247	9.1	72.09	2.48	1814	16	2	JBHRKQ000000000	SRR28645819	No
788__bin.20	s__Corynebacterium matruchotii	s__Corynebacterium matruchotii (SGB17007)	2802685	57.21	173	24743	24.4	97.21	0.09	2582	45	3	JBHRKP000000000	SRR28645819	No
788__bin.25	s__Bacteroides_D sp013333835	s__GGB1025 SGB1319 (SGB1319)	2439212	55.33	175	20642	38.7	97.14	0.12	1844	37	0	JBHRKO000000000	SRR28645819	No
788__bin.26	g__CAJPQU01	s__GGB4299 SGB5872 (SGB5872)	1050880	54.93	398	2710	6.8	55.35	0.65	1040	8	0	JBHRKN000000000	SRR28645819	No
788__bin.27	s__Centipeda noxia	s__Selenomonas noxia (SGB5884)	1805488	56.54	92	32567	42.6	78.28	0.92	1736	53	3	JBHRKM000000000	SRR28645819	No
788__bin.28	s__Tannerella forsythia	s__Tannerella forsythia (SGB2053)	2860719	47.87	192	22993	17.2	97.37	0.58	2396	41	1	JBHRKL000000000	SRR28645819	No
788__bin.29	s__Prevotella denticola	s__Prevotella denticola (SGB1540)	2006612	51.63	549	4138	9.6	54.62	1.72	1660	24	0	JBHRKK000000000	SRR28645819	No
788__bin.34	s__F0058 sp000163695	s__Bacteroidetes oral taxon 274 (SGB1341)	1979199	43.45	160	17974	23.7	96.77	0.81	1749	40	2	JBHRKJ000000000	SRR28645819	No
788__bin.4	g__Actinomyces	NA	1911280	68.49	758	2502	29.6	51.26	2.96	1886	28	1	JBHRKI000000000	SRR28645819	No
788__bin.5	s__Veillonella parvula_A	s__Veillonella parvula (SGB6939)	1286912	39.71	413	3363	11.5	73.20	2.02	1083	5	0	JBHRKH000000000	SRR28645819	No
788__bin.6	s__Prevotella sp013333935	s__GGB1202 SGB1566 (SGB1566)	1910125	49.11	458	4709	8.8	74.56	1.32	1578	22	1	JBHRKG000000000	SRR28645819	No
788__bin.7	s__Treponema_D buccale	s__Treponema socranskii (SGB3585)	1975684	50.38	584	3808	10.4	73.38	0.68	1800	21	1	JBHRKF000000000	SRR28645819	No
791__bin.11	s__Porphyromonas endodontalis	s__Porphyromonas endodontalis (SGB2016)	1636292	48.08	221	9516	12.4	90.55	1.57	1351	36	1	JBHRKE000000000	SRR28645818	No
791__bin.13	g__JABCPE02	NA	1459595	37.96	283	6228	36.0	91.64	4.84	1323	24	2	JBHRKD000000000	SRR28645818	No
791__bin.14	s__Prevotella maculosa	s__Prevotella maculosa (SGB1524)	2552529	48.25	228	14077	40.7	91.00	3.56	2085	47	3	JBHRKC000000000	SRR28645818	No
791__bin.15	s__Prevotella salivae	s__Prevotella salivae (SGB1522)	2085891	41.74	580	3946	18.4	62.51	3.83	1563	23	0	JBHRKB000000000	SRR28645818	No
791__bin.16	g__Catonella	s__Catonella SGB4505 (SGB4505)	1532534	37.82	525	3107	12.5	63.48	2.57	1259	9	0	JBHRKA000000000	SRR28645818	No
791__bin.19	g__Prevotella	NA	3422331	47.61	438	10094	117.6	91.55	4.55	2694	44	1	JBHRJZ000000000	SRR28645818	No
791__bin.20	s__HOT-345 sp003260355	s__SR1 bacterium human oral taxon HOT 345 (SGB6893)	1082466	38.23	43	32654	72.1	70.22	2.25	2002	39	2	JBHRJY000000000	SRR28645818	No
791__bin.22	s__Lachnoanaerobaculum gingivalis	s__Lachnoanaerobaculum gingivalis (SGB4493)	2532682	36.59	851	3167	26.1	63.79	4.31	2197	20	0	JBHRJX000000000	SRR28645818	No
791__bin.24	s__Prevotella intermedia	s__Prevotella intermedia (SGB1560)	2056392	44.25	367	6838	24.0	88.10	0.34	1674	25	0	JBHRJW000000000	SRR28645818	No
791__bin.25	s__Alloprevotella sp003639005	s__Alloprevotella sp Lung230 (SGB1467)	1895468	54.52	203	11374	22.2	81.42	0.49	1522	38	1	JBHRJV000000000	SRR28645818	No
791__bin.26	s__SDRW01 sp007845485	s__TM7 phylum sp oral taxon 348 (SGB19880)	786798	38.26	85	14310	24.3	61.68	1.71	755	38	1	JBHRJU000000000	SRR28645818	No
791__bin.28	s__Haemophilus_D parainfluenzae	s__Haemophilus parainfluenzae (SGB9712)	2037034	39.33	93	32968	69.0	98.77	2.10	1928	47	5	JBHRJT000000000	SRR28645818	No
791__bin.30	s__Prevotella melaninogenica_B	NA	2942558	41.19	210	21016	178.9	94.93	3.48	2288	37	3	JBHRJS000000000	SRR28645818	No
791__bin.35	s__Capnocytophaga leadbetteri	NA	1467579	41.23	158	13168	111.0	58.61	0.95	1301	27	0	JBHRJR000000000	SRR28645818	No
791__bin.36	s__Neisseria subflava	s__Neisseria subflava (SGB9450_group)	1452723	49.66	356	4761	15.3	68.97	2.32	1554	26	1	JBHRJQ000000000	SRR28645818	No
791__bin.37	s__Saccharimonas sp013333845	s__Candidatus Nanosynsacchari sp TM7 ANC 38 39 G1 1 (SGB19882)	787235	48.20	38	34235	24.3	64.51	1.08	778	44	2	JBHRJP000000000	SRR28645818	No
791__bin.38	s__Dialister invisus	s__Dialister SGB47054 (SGB47054_group)	1525574	45.74	300	6305	14.3	88.52	2.20	1442	38	1	JBHRJO000000000	SRR28645818	No
791__bin.41	s__Peptidiphaga sp000466165	s__Actinobaculum sp oral taxon 183 (SGB15892)	2180913	68.05	224	13632	19.6	87.92	3.42	1876	49	0	JBHRJN000000000	SRR28645818	No
791__bin.42	s__F0058 sp000163695	s__Bacteroidetes oral taxon 274 (SGB1341)	1154830	42.55	211	6979	25.5	61.81	0.00	1055	22	1	JBHRJM000000000	SRR28645818	No
791__bin.46	s__Neisseria elongata	s__Neisseria elongata (SGB9447)	2156095	54.44	180	16917	36.6	91.76	3.76	2107	44	1	JBHRJL000000000	SRR28645818	No
791__bin.5	s__Veillonella parvula_A	s__Veillonella parvula (SGB6939)	1278977	38.30	289	4764	194.3	71.24	3.89	1161	28	3	JBHRJK000000000	SRR28645818	No
791__bin.50	s__Anaeroglobus micronuciformis	s__Megasphaera micronuciformis (SGB5868)	1298862	46.06	253	6339	11.5	83.97	1.57	1216	26	0	JBHRJJ000000000	SRR28645818	No
791__bin.51	g__Campylobacter_A	s__Campylobacter SGB19316 (SGB19316_group)	2267582	45.81	131	29046	109.9	98.21	4.51	2136	38	0	JBHRJI000000000	SRR28645818	No
791__bin.53	s__Fusobacterium polymorphum	s__Fusobacterium nucleatum (SGB6001)	2483168	27.39	263	14863	214.9	94.94	2.86	2470	32	4	JBHRJH000000000	SRR28645818	No
791__bin.54	s__Bacteroides_D sp905373475	s__GGB1022 SGB1315 (SGB1315)	2332598	46.74	209	17135	19.2	93.08	0.71	1753	34	2	JBHRJG000000000	SRR28645818	No
791__bin.55	s__Treponema_C maltophilum	s__Treponema maltophilum (SGB3589)	1310813	49.14	495	2731	10.1	59.95	2.68	1250	22	0	JBHRJF000000000	SRR28645818	No
791__bin.56	s__Aggregatibacter sp000466335	s__Aggregatibacter sp 2125159857 (SGB9733)	1880864	42.97	86	32859	113.0	97.47	1.45	1746	45	6	JBHRJE000000000	SRR28645818	No
791__bin.57	s__Porphyromonas sp000467855	s__Porphyromonas sp oral taxon 278 (SGB2041)	1819571	56.07	243	9465	47.1	64.11	0.00	1411	28	0	JBHRJD000000000	SRR28645818	No
791__bin.61	s__F0058 sp905372605	s__Bacteroidota unclassified SGB1343 (SGB1343)	1789336	43.55	144	15864	64.6	95.43	3.05	1518	46	2	JBHRJC000000000	SRR28645818	No
791__bin.62	s__Eikenella corrodens	s__Eikenella corrodens (SGB9434_group)	1561898	56.42	384	4763	17.3	67.18	1.72	1640	36	0	JBHRJB000000000	SRR28645818	No
791__bin.64	g_Nanosyncoccus	s__GGB12761 SGB19791 (SGB19791)	683286	38.50	23	44453	51.4	80.88	0.00	629	44	3	JBHRJA000000000	SRR28645818	No
791__bin.65	s__Bacteroides_D sp013333835	s__GGB1025 SGB1319 (SGB1319)	2292642	55.20	189	18253	24.1	92.14	0.48	1745	38	0	JBHRIZ000000000	SRR28645818	No
791__bin.66	s__Porphyromonas pasteri	s__Porphyromonas bobii (SGB2043)	1647890	56.41	64	39906	130.5	70.41	2.32	1297	36	3	JBHRIY000000000	SRR28645818	No
791__bin.68	s__Corynebacterium matruchotii	s__Corynebacterium matruchotii (SGB17007)	2432781	57.82	237	13654	38.7	86.74	2.41	2282	48	3	JBHRIX000000000	SRR28645818	No
791__bin.7	s__Kingella_B oralis	s__Kingella bonacorsii (SGB9422)	1928348	54.95	316	8029	20.9	85.27	2.21	1891	38	0	JBHRIW000000000	SRR28645818	No
791__bin.70	s__Alloprevotella tannerae	s__Alloprevotella SGB77721 (SGB77721_group)	1640792	48.56	168	11715	17.8	62.07	0.00	1289	27	1	JBHRIV000000000	SRR28645818	No
791__bin.71	s__Prevotella oulorum	s__Prevotella oulorum (SGB1520)	1979020	47.63	164	14521	48.5	84.94	0.51	1638	36	1	JBHRIU000000000	SRR28645818	No
791__bin.72	s__Prevotella saccharolytica	s__Prevotella saccharolytica (SGB1454)	2051209	43.58	482	5051	15.3	77.69	1.65	1586	24	0	JBHRIT000000000	SRR28645818	No
791__bin.75	g__Cardiobacterium	s__Cardiobacterium SGB9419 (SGB9419)	1102778	58.30	242	5169	93.4	50.00	0.00	1156	21	0	JBHRIS000000000	SRR28645818	No
809__bin.1	s__Prevotella nigrescens	s__Prevotella nigrescens (SGB1561)	1668905	44.53	179	11856	16.4	68.68	0.34	1350	17	1	JBHRIR000000000	SRR28645817	Yes
809__bin.10	s__Corynebacterium matruchotii	s__Corynebacterium matruchotii (SGB17007)	2823160	57.25	74	72168	119.8	98.96	0.06	2525	46	5	JBHRIQ000000000	SRR28645817	Yes
809__bin.12	s__Streptococcus sobrinus	s__Streptococcus sobrinus (SGB7995)	2050318	43.35	91	37869	50.4	93.92	2.62	1946	22	0	JBHRIP000000000	SRR28645817	Yes
809__bin.13	s__Limosilactobacillus fermentum	s__Limosilactobacillus fermentum (SGB7106)	1568964	53.70	240	9289	13.4	92.71	2.04	1537	29	2	JBHRIO000000000	SRR28645817	Yes
809__bin.16	s__Veillonella atypica	s__Veillonella atypica (SGB6936)	1541784	40.11	113	18916	90.5	70.99	4.47	1402	27	4	JBHRIN000000000	SRR28645817	Yes
809__bin.19	s__Bacteroides_D sp905373475	s__GGB1022 SGB1315 (SGB1315)	2342254	46.60	103	37608	27.9	95.48	0.00	1743	39	2	JBHRIM000000000	SRR28645817	Yes
809__bin.20	s__Capnocytophaga gingivalis	s__Capnocytophaga gingivalis (SGB2479)	2512547	40.25	199	20536	97.6	95.82	0.24	2375	42	2	JBHRIL000000000	SRR28645817	Yes
809__bin.23	s__Rothia dentocariosa	s__Rothia SGB49305 (SGB49305_group)	1674771	55.00	58	44888	80.8	61.26	0.00	1456	26	3	JBHRIK000000000	SRR28645817	Yes
809__bin.25	s__Parascardovia denticolens	s__Parascardovia denticolens (SGB17291)	1616284	59.32	166	13754	13.0	96.27	1.25	1294	39	0	JBHRIJ000000000	SRR28645817	Yes
809__bin.28	s__Streptococcus gordonii	s__Streptococcus gordonii (SGB8053)	1881792	40.86	60	50370	69.8	86.67	1.43	1800	22	1	JBHRII000000000	SRR28645817	Yes
809__bin.29	s__Prevotella oris	s__Prevotella oris (SGB1525)	2877851	43.95	268	16691	19.2	96.34	2.13	2397	41	1	JBHRIH000000000	SRR28645817	Yes
809__bin.3	s__Centipeda artemidis	s__Selenomonas artemidis (SGB5878)	1381836	58.33	446	3226	10.9	66.95	3.71	1419	16	0	JBHRIG000000000	SRR28645817	Yes
809__bin.30	s__Prevotella histicola	s__Prevotella histicola (SGB1543)	2422909	41.73	499	5769	12.8	92.36	0.69	1911	33	0	JBHRIF000000000	SRR28645817	Yes
809__bin.6	s__Centipeda noxia	s__Selenomonas noxia (SGB5884)	1794283	56.40	118	24327	14.7	82.35	2.04	1701	42	1	JBHRIE000000000	SRR28645817	Yes
809__bin.7	s__Streptococcus mutans	s__Streptococcus mutans (SGB8000)	1740982	37.13	286	8719	18.3	89.85	0.58	1601	9	0	JBHRID000000000	SRR28645817	Yes
809__bin.8	s__Scardovia wiggsiae	s__Scardovia wiggsiae (SGB15494)	1406361	53.30	155	13407	11.0	93.25	0.64	1162	38	0	JBHRIC000000000	SRR28645817	Yes
809__bin.9	s__Eikenella halliae	s__Eikenella halliae (SGB9430)	2230701	56.76	111	30630	79.7	93.10	1.66	2122	43	1	JBHRIB000000000	SRR28645817	Yes
813__bin.12	s__Capnocytophaga granulosa	s__Capnocytophaga granulosa (SGB2483)	1991259	42.82	456	4997	12.3	86.96	1.11	1706	31	2	JBHRIA000000000	SRR28645816	Yes
813__bin.13	s__Veillonella parvula_A	s__Veillonella parvula (SGB6939)	1382186	39.17	381	3925	20.1	78.60	2.64	1233	17	0	JBHRHZ000000000	SRR28645816	Yes
813__bin.14	s__Centipeda noxia	NA	761697	53.43	28	39795	20.1	51.72	1.72	754	20	2	JBHRHY000000000	SRR28645816	Yes
813__bin.17	g__Campylobacter_A	NA	2006699	46.18	177	14911	14.2	99.19	1.61	1931	32	1	JBHRHX000000000	SRR28645816	Yes
813__bin.2	s__Rothia aeria	s__Rothia aeria (SGB16987)	2111516	57.28	192	17168	65.6	83.76	1.26	1852	42	2	JBHRHW000000000	SRR28645816	Yes
813__bin.22	s__Haemophilus_D parainfluenzae	s__Haemophilus parainfluenzae (SGB9712)	1333135	39.83	450	3058	19.2	65.31	3.12	1267	11	0	JBHRHV000000000	SRR28645816	Yes
813__bin.3	s__Rothia dentocariosa	s__Rothia SGB49305 (SGB49305_group)	1582069	55.50	95	29945	120.2	52.65	0.66	1426	26	1	JBHRHU000000000	SRR28645816	Yes
813__bin.7	s__Corynebacterium matruchotii	s__Corynebacterium matruchotii (SGB17007)	2155702	58.01	73	43889	61.1	73.68	0.00	1962	40	3	JBHRHT000000000	SRR28645816	Yes
813__bin.9	s__Haemophilus seminalis	s__Haemophilus haemolyticus (SGB9649)	1888379	38.33	115	26478	31.0	98.43	1.87	1801	45	5	JBHRHS000000000	SRR28645816	Yes
817__bin.1	g__Lancefieldella	NA	1096750	49.81	277	4382	7.0	67.54	1.32	992	20	0	JBHRHR000000000	SRR28645814	Yes
817__bin.10	s__Capnocytophaga ochracea	s__Capnocytophaga ochracea (SGB2497)	2167882	40.17	554	4471	11.6	86.28	1.44	1768	19	1	JBHRHQ000000000	SRR28645814	Yes
817__bin.13	s__CAJPQU01 sp905373705	s__GGB4299 SGB5872 (SGB5872)	1891807	55.30	155	18787	26.7	94.68	4.19	1809	38	4	JBHRHP000000000	SRR28645814	Yes
817__bin.16	s__Saccharimonas sp013333795	NA	789517	48.30	28	40368	17.9	61.27	0.93	792	38	2	JBHRHO000000000	SRR28645814	Yes
817__bin.17	s__Porphyromonas endodontalis	s__Porphyromonas endodontalis (SGB2016)	1805238	47.75	145	18986	10.5	98.19	0.79	1491	41	0	JBHRHN000000000	SRR28645814	Yes
817__bin.19	g__Nanoperiomorbus	NA	638980	51.39	26	32772	237.1	53.65	0.85	561	37	2	JBHRHM000000000	SRR28645814	Yes
817__bin.20	s__CAJPQU01 sp905372875	s__GGB97311 SGB5873 (SGB5873)	1947865	55.15	84	32046	45.9	98.76	0.32	1829	41	2	JBHRHL000000000	SRR28645814	Yes
817__bin.22	s__Actinomyces dentalis	s__Actinomyces dentalis (SGB15877)	2690503	72.36	890	3224	15.9	69.61	3.74	2487	40	0	JBHRHK000000000	SRR28645814	Yes
817__bin.23	s__Prevotella sp013333935	s__GGB1202 SGB1566 (SGB1566)	2244343	48.70	268	11246	49.0	89.10	3.49	1853	35	2	JBHRHJ000000000	SRR28645814	Yes
817__bin.26	s__Scardovia wiggsiae	s__Scardovia wiggsiae (SGB15494)	784463	53.74	276	2948	6.0	52.61	0.45	745	24	0	JBHRHI000000000	SRR28645814	Yes
817__bin.29	s__Treponema_D buccale	s__Treponema socranskii (SGB3585)	2365349	50.05	447	6659	12.3	96.81	2.57	2085	39	0	JBHRHH000000000	SRR28645814	Yes
817__bin.33	s__Saccharimonas sp905371715	s__Candidatus Saccharibacteria unclassified SGB19846 (SGB19846_group)	667549	49.84	31	33669	15.3	57.41	0.00	658	35	1	JBHRHG000000000	SRR28645814	Yes
817__bin.34	s__Prevotella oulorum	s__Prevotella oulorum (SGB1520)	2159814	47.85	222	12119	51.9	90.15	3.06	1776	39	1	JBHRHF000000000	SRR28645814	Yes
817__bin.36	s__Prevotella histicola	s__Prevotella histicola (SGB1543)	1902933	42.45	271	9352	47.5	65.06	1.24	1463	24	2	JBHRHE000000000	SRR28645814	Yes
817__bin.39	s__Prevotella nigrescens	s__Prevotella nigrescens (SGB1561)	2123929	43.11	133	26017	46.0	74.29	3.45	1744	34	0	JBHRHD000000000	SRR28645814	Yes
817__bin.7	s__Olsenella_F sp001189515	s__Olsenella sp oral taxon 807 (SGB14348)	2752008	61.41	325	11860	15.7	99.66	2.16	2457	43	0	JBHRHC000000000	SRR28645814	Yes
821__bin.10	s__Tannerella forsythia	s__Tannerella forsythia (SGB2053)	2830859	47.88	204	21555	32.8	94.36	0.33	2304	38	2	JBHRHB000000000	SRR28645813	No
821__bin.16	s__Bacteroides_D sp905373475	s__GGB1022 SGB1315 (SGB1315)	2477794	46.57	86	47432	126.8	96.19	0.73	1854	39	5	JBHRHA000000000	SRR28645813	No
821__bin.18	g__Treponema_D	s__GGB2666 SGB69449 (SGB69449)	2314977	42.94	269	12238	16.9	97.20	0.00	1873	41	2	JBHRGZ000000000	SRR28645813	No
821__bin.20	s__SDRK01 sp007845205	s__TM7 phylum sp oral taxon 350 (SGB19820)	558416	34.84	136	4799	14.1	66.15	0.00	492	21	1	JBHRGY000000000	SRR28645813	No
821__bin.22	s__Prevotella nigrescens	s__Prevotella nigrescens (SGB1561)	2701530	42.19	236	17983	65.0	99.15	3.07	2351	33	0	JBHRGX000000000	SRR28645813	No
821__bin.24	s__Treponema_D buccale	s__Treponema socranskii (SGB3585)	2267420	49.71	748	3290	16.3	66.94	4.15	2143	30	0	JBHRGW000000000	SRR28645813	No
821__bin.27	s__Treponema_C lecithinolyticum	s__GGB2663 SGB3587 (SGB3587)	2120664	44.07	101	37290	25.9	99.30	0.51	1848	43	1	JBHRGV000000000	SRR28645813	No
821__bin.28	s__Capnocytophaga granulosa	s__Capnocytophaga granulosa (SGB2483)	1082160	44.53	390	2888	10.0	53.61	1.43	835	8	0	JBHRGU000000000	SRR28645813	No
821__bin.31	s__Prevotella denticola	s__Prevotella denticola (SGB1540)	2044544	51.74	524	4489	9.6	76.03	1.68	1683	18	1	JBHRGT000000000	SRR28645813	No
821__bin.32	s__Centipeda massiliensis	s__Selenomonas massiliensis (SGB5886)	2445142	56.30	578	5268	34.3	61.71	4.31	2475	32	1	JBHRGS000000000	SRR28645813	No
821__bin.33	s__Porphyromonas endodontalis	NA	1411480	48.23	388	3987	7.3	75.10	1.34	1150	22	0	JBHRGR000000000	SRR28645813	No
821__bin.36	s__Prevotella conceptionensis	NA	3236170	47.92	700	5442	30.5	88.40	4.61	2573	38	3	JBHRGQ000000000	SRR28645813	No
821__bin.4	s__Prevotella sp013333935	s__GGB1202 SGB1566 (SGB1566)	1453367	49.79	428	3821	9.1	55.69	1.25	1193	15	0	JBHRGP000000000	SRR28645813	No
821__bin.42	s__F0058 sp000163695	s__Bacteroidetes oral taxon 274 (SGB1341)	2124914	42.99	127	27009	80.4	97.31	2.69	1914	41	2	JBHRGO000000000	SRR28645813	No
827__bin.10	s__Prevotella nigrescens	s__Prevotella nigrescens (SGB1561)	1959305	43.13	102	29169	35.1	67.24	1.72	1641	31	1	JBHRGN000000000	SRR28645812	No
827__bin.11	s__Prevotella denticola	s__Prevotella denticola (SGB1540)	2431999	50.68	133	27147	52.3	89.97	0.45	1967	43	3	JBHRGM000000000	SRR28645812	No
827__bin.13	s__Rothia aeria	s__Rothia aeria (SGB16987)	1511713	57.53	187	10560	48.1	55.50	0.00	1339	29	0	JBHRGL000000000	SRR28645812	No
827__bin.14	s__Haemophilus_D parainfluenzae	s__Haemophilus parainfluenzae (SGB9712)	1921721	39.39	107	27942	20.0	94.35	0.07	1753	43	3	JBHRGK000000000	SRR28645812	No
827__bin.16	s__Capnocytophaga gingivalis	s__Capnocytophaga gingivalis (SGB2479)	1535564	41.41	385	4625	10.3	54.23	0.00	1348	15	0	JBHRGJ000000000	SRR28645812	No
827__bin.2	s__Neisseria elongata	s__Neisseria elongata (SGB9447)	1885111	56.02	426	5383	210.7	58.62	4.31	1966	28	2	JBHRGI000000000	SRR28645812	No
827__bin.6	s__Cardiobacterium hominis	s__Cardiobacterium hominis (SGB9418)	1826265	60.08	355	6503	15.4	65.52	0.00	1793	38	0	JBHRGH000000000	SRR28645812	No
827__bin.7	s__Peptidiphaga sp000466165	s__Actinobaculum sp oral taxon 183 (SGB15892)	1801323	68.33	338	7011	46.3	72.88	0.42	1623	25	0	JBHRGG000000000	SRR28645812	No
827__bin.9	s__Corynebacterium matruchotii	s__Corynebacterium matruchotii (SGB17007)	2265424	58.65	294	10442	862.0	73.80	2.98	2160	38	3	JBHRGF000000000	SRR28645812	No
830__bin.11	s__Capnocytophaga ochracea	s__Capnocytophaga ochracea (SGB2497)	2424358	39.76	461	6496	30.2	93.79	4.43	2069	33	2	JBHRGE000000000	SRR28645811	No
830__bin.14	s__Rothia dentocariosa	s__Rothia SGB49305 (SGB49305_group)	1692373	52.78	320	6370	33.7	76.05	2.92	1540	42	0	JBHRGD000000000	SRR28645811	No
830__bin.18	s__Prevotella oulorum	s__Prevotella oulorum (SGB1520)	2180021	47.74	210	14342	14.1	90.90	0.90	1835	39	2	JBHRGC000000000	SRR28645811	No
830__bin.19	s__Centipeda artemidis	s__Selenomonas artemidis (SGB5878)	2032785	57.65	139	21785	38.7	95.97	2.67	1890	36	3	JBHRGB000000000	SRR28645811	No
830__bin.21	s__Lachnoanaerobaculum saburreum	s__Lachnoanaerobaculum saburreum (SGB4481)	1667662	37.69	589	2967	7.8	58.61	1.59	1455	17	0	JBHRGA000000000	SRR28645811	No
830__bin.22	s__Selenomonas sp905372355	s__Selenomonas SGB5897 (SGB5897_group)	1957868	57.78	316	8498	12.0	86.65	0.00	1807	29	1	JBHRFZ000000000	SRR28645811	No
830__bin.25	s__Kingella_B oralis	s__Kingella bonacorsii (SGB9422)	2034489	54.31	393	6862	11.7	86.60	2.03	1989	34	0	JBHRFY000000000	SRR28645811	No
830__bin.28	s__Centipeda noxia	s__Selenomonas noxia (SGB5884)	1389061	54.60	37	62513	71.3	81.84	1.56	1361	34	2	JBHRFX000000000	SRR28645811	No
830__bin.29	s__Prevotella nigrescens	s__Prevotella nigrescens (SGB1561)	2448300	42.28	117	37388	49.9	96.95	0.19	2089	38	1	JBHRFW000000000	SRR28645811	No
830__bin.6	s__Corynebacterium matruchotii	s__Corynebacterium matruchotii (SGB17007)	2838784	57.23	94	53373	77.6	93.66	0.44	2584	57	6	JBHRFV000000000	SRR28645811	No
830__bin.7	s__Cardiobacterium hominis	s__Cardiobacterium hominis (SGB9418)	2095369	59.92	504	4818	10.6	68.97	1.72	2078	40	0	JBHRFU000000000	SRR28645811	No
830__bin.9	s__Leptotrichia hongkongensis	s__Leptotrichia hongkongensis (SGB6059)	1735744	30.91	494	3781	24.9	86.93	2.96	1550	13	0	JBHRFT000000000	SRR28645811	No

^*a*^ Accession numbers for assemblies and respective raw metagenomic reads are also provided. NAs are included for the PhyloPhlAn classification when the MAG did not belong (i.e., it had <95% average nucleotide identity) to any species-level genome bin (SGB) in the vJun23 PhyloPhlAn database.

## Data Availability

Metagenome-assembled genomes have been deposited in GenBank under the accession numbers listed in Table 1. Metagenomic reads have been deposited in NCBI-SRA under the accession no. PRJNA1093152.
